# (*E*)-2-Acetyl-4-[(3-methyl­phenyl­)diazen­yl]phenol: an X-ray and DFT study

**DOI:** 10.1107/S1600536810003491

**Published:** 2010-02-06

**Authors:** Serap Yazıcı, Çiğdem Albayrak, İsmail Gümrükçüoğlu, İsmet Şenel, Orhan Büyükgüngör

**Affiliations:** a Department of Physics, Faculty of Arts and Sciences, Ondokuz Mayıs University, TR-55139 Kurupelit-Samsun, Turkey; b Sinop University, Sinop Faculty of Education, TR-57000 Sinop, Turkey; c Department of Chemistry, Ondokuz Mayıs University, TR-55139 Kurupelit-Samsun, Turkey

## Abstract

The title compound, C_15_H_14_N_2_O_2_, an azo dye, displays a *trans* configuration with respect to the N=N bridge. The dihedral angle between the aromatic rings is 0.18 (14)°. There is a strong intra­molecular O—H⋯O hydrogen bond. Geometrical parameters, determined using X-ray diffraction techniques, are compared with those calculated by density functional theory (DFT), using hybrid exchange–correlation functional, B3LYP and semi-empirical (PM3) methods.

## Related literature

For general background to azo compounds, see: Klaus (2003 ▶); Catino & Farris (1985 ▶); Zollinger (2003 ▶); Bahatti & Seshadri (2004 ▶); Taniike *et al.* (1996 ▶); Fadda *et al.* (1994 ▶). For a related structure, see: El-Ghamry *et al.* (2008 ▶). For background to DFT calculations, see: Becke (1988 ▶, 1993 ▶); Lee *et al.* (1988 ▶); Schmidt & Polik (2007 ▶)
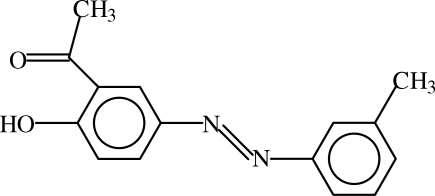


## Experimental

### 

#### Crystal data

C_15_H_14_N_2_O_2_
*M* *_r_* = 254.28
Monoclinic, 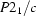

*a* = 8.6917 (3) Å
*b* = 10.9728 (3) Å
*c* = 14.6150 (5) Å
β = 112.881 (3)°
*V* = 1284.19 (7) Å^3^
*Z* = 4
Mo *K*α radiation
μ = 0.09 mm^−1^
*T* = 150 K
0.67 × 0.37 × 0.21 mm

#### Data collection

Stoe IPDS II diffractometer
Absorption correction: integration (*X-RED32*; Stoe & Cie, 2002 ▶) *T*_min_ = 0.957, *T*_max_ = 0.986
16525 measured reflections
2519 independent reflections
2034 reflections with *I* > 2σ(*I*)
*R*_int_ = 0.040

#### Refinement

*R*[*F* ^2^ > 2σ(*F* ^2^)] = 0.060
*wR*(*F* ^2^) = 0.175
*S* = 1.06
2519 reflections
176 parameters
H atoms treated by a mixture of independent and constrained refinement
Δρ_max_ = 0.48 e Å^−3^
Δρ_min_ = −0.26 e Å^−3^

### 

Data collection: *X-AREA* (Stoe & Cie, 2002 ▶); cell refinement: *X-AREA*; data reduction: *X-RED32* (Stoe & Cie, 2002 ▶); program(s) used to solve structure: *SHELXS97* (Sheldrick, 2008 ▶); program(s) used to refine structure: *SHELXL97* (Sheldrick, 2008 ▶); molecular graphics: *ORTEP-3 for Windows* (Farrugia, 1997 ▶); software used to prepare material for publication: *WinGX* (Farrugia, 1999 ▶) and *GAUSSIAN* (Frisch *et al.*, 2004 ▶).

## Supplementary Material

Crystal structure: contains datablocks I, global. DOI: 10.1107/S1600536810003491/bt5181sup1.cif

Structure factors: contains datablocks I. DOI: 10.1107/S1600536810003491/bt5181Isup2.hkl

Additional supplementary materials:  crystallographic information; 3D view; checkCIF report

## Figures and Tables

**Table 1:** Hydrogen-bond geometry (Å, °)

*D*—H⋯*A*	*D*—H	H⋯*A*	*D*⋯*A*	*D*—H⋯*A*
O1—H1⋯O2	0.84 (4)	1.78 (4)	2.567 (3)	156 (4)

**Table 2:** Selected geometric parameters (Å, °) calculated with X-ray, PM3 and DFT

Parameters	X-ray	PM3	DFT/B3LYP*
C4—O1	1.343 (3)	1.351	1.331
C7—O2	1.235 (3)	1.228	1.242
C7—C8	1.488 (3)	1.502	1.513
C13—C15	1.493 (4)	1.486	1.511
C1—N2	1.444 (3)	1.445	1.411
N1—N2	1.242 (3)	1.232	1.263
C9—N1	1.450 (3)	1.447	1.417
O2—C7—C8	119.8 (2)	120.465	118.986
O1—C4—C5	117.2 (2)	115.387	118.123
C7—C3—C4—O1	1.7 (3)	−0.016	0.002
C9—N1—N2—C1	−179.99 (17)	−179.965	−179.975
C2—C1—N2—N1	177.09 (19)	−178.543	179.996
C10—C9—N1—N2	−177.6 (2)	−172.651	179.997

*6–31G(d,p).

**Fig. 1.**
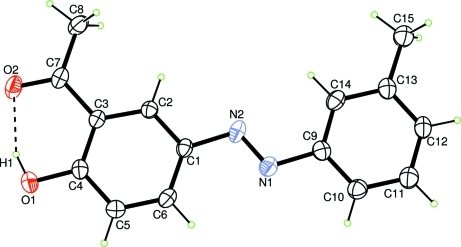
A view of the title compound with the atomic numbering scheme. Displacement ellipsoids are drawn at the 30% probability level. The dashed line indicates the intramolecular hydrogen bond.

## supplementary crystallographic information

### Comment

Azo compounds are very important in the field of dyes, pigments and advanced materials (Klaus, 2003). It has been known for many years that the azo compounds are the most widely used class of dyes, due to their versatile applications in various fields such as the dyeing of textile fibers, the coloring of different materials, colored plastics and polymers, biological-medical studies and advanced applications in organic synthesis (Bahatti & Seshadri, 2004; Catino & Farris, 1985; Fadda *et al.*, 1994; Taniike *et al.*, 1996; Zollinger, 2003).

In the title compound, C_15_H_14_N_2_O_2_, the two aromatic groups atteched to the azo bridge are adopted (E) configuration. The molecule is planar and the dihedral angle between the two aromatic rings is 0.18(0.14)°. All the bond lengths are in agreement with reported for other azo compounds (El-Ghamry *et al.*, 2008). The title molecule (Fig. 1) has a  strong intramolecular hydrogen bond between the hydroxyl group and the carbonyl O atom.

Density-functional theory (DFT) (Schmidt & Polik, 2007) and semi-empirical (PM3) calculations and full-geometry optimizations were performed by means of *GAUSSIAN* 03 W package (Frisch *et al.*, 2004). The selected bond lengths and angles (Table 2.) obtained from semi-empirical and DFT/B3LYP (Becke, 1988; Becke 1993; Lee *et al.* 1988) are given in Table 2. As can be seen Table 2. the bond lenghts and angles achieved by DFT method are better than those values obtained from PM3 method.

### Experimental

A mixture of 3-methylaniline (0.83 g, 7.8 mmol), water (20 ml) and concentrated hydrochloric acid (1.97 ml, 23.4 mmol) was stirred until a clear solution was obtained. This solution was cooled down to 0–5 °C and a solution of sodium nitrite (0.75 g 7.8 mmol) in water was added dropwise while the temperature was maintained below 5 °C. The resulting mixture was stirred for 30 min in an ice bath. 2-hydroxyacetophenone (1.067 g, 7.8 mmol solution (pH 9) was gradually added to a cooled solution of 3-methylbenzenediazonium chloride, prepared as described above, and the resulting mixture was stirred at 0–5 °C for 2 h in ice bath. The product was recrystallized from ethyl alcohol to obtain solid (*E*)-2-Acetyl-4- (3-methylphenyldiazenyl)phenol. Crystals of (*E*)-2-Acetyl-4-(3-methylphenyldiazenyl)phenol were obtained after one day by slow evaporation from acetic acid (yield %45, m.p.= 377–379 K)

### Refinement

All H atoms (except for H1) were placed in calculated positions and constrained to ride on their parents atoms, with C—H = 0.93–0.97 Å, O—H = 0.98 Å and *U*_iso_(H) = 1.2*U*_eq_(C) or 1.5*U*_eq_(C). The hydroxyl H atom was isotropically refined.

### Crystal data

**Table:**

C_15_H_14_N_2_O_2_	*F*(000) = 536
*M**_r_* = 254.28	*D*_x_ = 1.315 Mg m^−^^3^
Monoclinic, *P*2_1_/*c*	Mo *K*α radiation, λ = 0.71073 Å
Hall symbol: -P 2ybc	Cell parameters from 20945 reflections
*a* = 8.6917 (3) Å	θ = 1.9–28.0°
*b* = 10.9728 (3) Å	µ = 0.09 mm^−^^1^
*c* = 14.6150 (5) Å	*T* = 150 K
β = 112.881 (3)°	Prism, brown
*V* = 1284.19 (7) Å^3^	0.67 × 0.37 × 0.21 mm
*Z* = 4	

### Data collection

**Table:**

Stoe IPDS II diffractometer	2519 independent reflections
Radiation source: fine-focus sealed tube	2034 reflections with *I* > 2σ(*I*)
graphite	*R*_int_ = 0.040
Detector resolution: 6.67 pixels mm^-1^	θ_max_ = 26.0°, θ_min_ = 2.4°
ω scan	*h* = −10→10
Absorption correction: integration (*X-RED32*; Stoe & Cie, 2002)	*k* = −13→13
*T*_min_ = 0.957, *T*_max_ = 0.986	*l* = −18→18
16525 measured reflections	

### Refinement

**Table:**

Refinement on *F*^2^	Primary atom site location: structure-invariant direct methods
Least-squares matrix: full	Secondary atom site location: difference Fourier map
*R*[*F*^2^ > 2σ(*F*^2^)] = 0.060	Hydrogen site location: inferred from neighbouring sites
*wR*(*F*^2^) = 0.175	H atoms treated by a mixture of independent and constrained refinement
*S* = 1.06	*w* = 1/[σ^2^(*F*_o_^2^) + (0.0859*P*)^2^ + 0.7485*P*] where *P* = (*F*_o_^2^ + 2*F*_c_^2^)/3
2519 reflections	(Δ/σ)_max_ < 0.001
176 parameters	Δρ_max_ = 0.48 e Å^−^^3^
0 restraints	Δρ_min_ = −0.26 e Å^−^^3^

### Special details

**Table:**

Experimental. 330 frames, detector distance = 80 mm
Geometry. All esds (except the esd in the dihedral angle between two l.s. planes) are estimated using the full covariance matrix. The cell esds are taken into account individually in the estimation of esds in distances, angles and torsion angles; correlations between esds in cell parameters are only used when they are defined by crystal symmetry. An approximate (isotropic) treatment of cell esds is used for estimating esds involving l.s. planes.
Refinement. Refinement of *F*^2^ against ALL reflections. The weighted *R*-factor wR and goodness of fit *S* are based on *F*^2^, conventional *R*-factors *R* are based on *F*, with *F* set to zero for negative *F*^2^. The threshold expression of *F*^2^ > σ(*F*^2^) is used only for calculating *R*-factors(gt) etc. and is not relevant to the choice of reflections for refinement. *R*-factors based on *F*^2^ are statistically about twice as large as those based on *F*, and *R*- factors based on ALL data will be even larger.

### Fractional atomic coordinates and isotropic or equivalent isotropic displacement parameters (Å^2^)

**Table:**

	*x*	*y*	*z*	*U*_iso_*/*U*_eq_	
C1	0.7697 (3)	0.49666 (19)	0.56898 (15)	0.0354 (5)	
C2	0.7798 (2)	0.48461 (19)	0.66522 (14)	0.0337 (5)	
H2	0.7188	0.5371	0.6882	0.040*	
C3	0.8796 (3)	0.39527 (19)	0.72861 (15)	0.0340 (5)	
C4	0.9690 (3)	0.3160 (2)	0.69197 (16)	0.0385 (5)	
C5	0.9567 (3)	0.3271 (2)	0.59383 (17)	0.0433 (5)	
H5	1.0150	0.2737	0.5695	0.052*	
C6	0.8588 (3)	0.4165 (2)	0.53369 (15)	0.0410 (5)	
H6	0.8517	0.4240	0.4688	0.049*	
C7	0.8963 (3)	0.3858 (2)	0.83278 (16)	0.0396 (5)	
C8	0.8087 (3)	0.4746 (3)	0.87272 (17)	0.0506 (6)	
H8A	0.8323	0.4560	0.9410	0.076*	
H8B	0.6905	0.4695	0.8350	0.076*	
H8C	0.8467	0.5556	0.8679	0.076*	
C9	0.5627 (3)	0.7083 (2)	0.37340 (17)	0.0392 (5)	
C10	0.5540 (3)	0.7208 (2)	0.27821 (17)	0.0460 (6)	
H10	0.6146	0.6692	0.2543	0.055*	
C11	0.4544 (3)	0.8107 (2)	0.21837 (18)	0.0482 (6)	
H11	0.4465	0.8197	0.1534	0.058*	
C12	0.3654 (3)	0.8882 (2)	0.25587 (17)	0.0447 (6)	
H12	0.2970	0.9479	0.2148	0.054*	
C13	0.3764 (3)	0.8785 (2)	0.35255 (17)	0.0426 (5)	
C14	0.4774 (3)	0.7866 (2)	0.41242 (16)	0.0426 (5)	
H14	0.4875	0.7778	0.4778	0.051*	
C15	0.2821 (3)	0.9642 (3)	0.39089 (19)	0.0546 (7)	
H15A	0.3037	0.9448	0.4588	0.082*	
H15B	0.1647	0.9566	0.3517	0.082*	
H15C	0.3171	1.0463	0.3868	0.082*	
N1	0.6655 (2)	0.60844 (18)	0.42943 (14)	0.0435 (5)	
N2	0.6666 (2)	0.59649 (18)	0.51418 (13)	0.0431 (5)	
O1	1.0685 (2)	0.22765 (17)	0.74768 (14)	0.0523 (5)	
O2	0.9858 (2)	0.30620 (17)	0.88764 (12)	0.0522 (5)	
H1	1.055 (4)	0.235 (3)	0.801 (3)	0.076 (11)*	

### Atomic displacement parameters (Å^2^)

**Table:**

	*U*^11^	*U*^22^	*U*^33^	*U*^12^	*U*^13^	*U*^23^
C1	0.0362 (10)	0.0347 (11)	0.0302 (10)	−0.0037 (8)	0.0074 (8)	0.0012 (8)
C2	0.0342 (10)	0.0327 (10)	0.0320 (10)	−0.0026 (8)	0.0106 (8)	−0.0006 (8)
C3	0.0347 (10)	0.0348 (11)	0.0299 (10)	−0.0039 (8)	0.0098 (8)	0.0018 (8)
C4	0.0386 (11)	0.0354 (11)	0.0376 (11)	0.0018 (9)	0.0104 (9)	0.0055 (9)
C5	0.0475 (13)	0.0427 (13)	0.0401 (12)	0.0041 (10)	0.0174 (10)	−0.0026 (10)
C6	0.0460 (12)	0.0458 (13)	0.0286 (10)	−0.0039 (10)	0.0118 (9)	0.0002 (9)
C7	0.0359 (11)	0.0473 (13)	0.0320 (10)	−0.0061 (10)	0.0091 (9)	0.0061 (9)
C8	0.0533 (14)	0.0671 (16)	0.0326 (11)	0.0004 (12)	0.0181 (10)	0.0003 (11)
C9	0.0378 (11)	0.0343 (11)	0.0432 (11)	−0.0041 (9)	0.0133 (9)	0.0002 (9)
C10	0.0511 (13)	0.0437 (13)	0.0426 (12)	−0.0030 (11)	0.0177 (11)	−0.0016 (10)
C11	0.0524 (14)	0.0438 (13)	0.0435 (12)	−0.0035 (11)	0.0133 (11)	0.0023 (10)
C12	0.0467 (12)	0.0375 (12)	0.0401 (12)	−0.0023 (10)	0.0062 (10)	0.0058 (9)
C13	0.0404 (12)	0.0379 (12)	0.0430 (12)	−0.0038 (10)	0.0093 (10)	0.0027 (10)
C14	0.0436 (12)	0.0471 (13)	0.0341 (11)	−0.0097 (10)	0.0119 (9)	0.0002 (9)
C15	0.0554 (15)	0.0574 (16)	0.0500 (14)	0.0057 (12)	0.0196 (12)	0.0049 (12)
N1	0.0464 (11)	0.0442 (11)	0.0375 (10)	−0.0024 (9)	0.0137 (8)	0.0008 (8)
N2	0.0430 (10)	0.0478 (11)	0.0309 (9)	−0.0097 (9)	0.0060 (8)	0.0069 (8)
O1	0.0587 (11)	0.0491 (10)	0.0484 (10)	0.0193 (8)	0.0201 (9)	0.0144 (8)
O2	0.0554 (10)	0.0611 (11)	0.0381 (8)	0.0056 (8)	0.0161 (8)	0.0179 (8)

### Geometric parameters (Å, °)

**Table:**

C1—C2	1.381 (3)	C9—C10	1.371 (3)
C1—C6	1.396 (3)	C9—C14	1.393 (3)
C1—N2	1.444 (3)	C9—N1	1.450 (3)
C2—C3	1.395 (3)	C10—C11	1.378 (3)
C2—H2	0.9300	C10—H10	0.9300
C3—C4	1.404 (3)	C11—C12	1.397 (4)
C3—C7	1.476 (3)	C11—H11	0.9300
C4—O1	1.343 (3)	C12—C13	1.383 (3)
C4—C5	1.402 (3)	C12—H12	0.9300
C5—C6	1.371 (3)	C13—C14	1.398 (3)
C5—H5	0.9300	C13—C15	1.493 (4)
C6—H6	0.9300	C14—H14	0.9300
C7—O2	1.235 (3)	C15—H15A	0.9600
C7—C8	1.488 (3)	C15—H15B	0.9600
C8—H8A	0.9600	C15—H15C	0.9600
C8—H8B	0.9600	N1—N2	1.242 (3)
C8—H8C	0.9600	O1—H1	0.83 (4)
			
C2—C1—C6	119.43 (19)	C10—C9—C14	121.7 (2)
C2—C1—N2	114.66 (19)	C10—C9—N1	115.3 (2)
C6—C1—N2	125.89 (19)	C14—C9—N1	123.0 (2)
C1—C2—C3	121.3 (2)	C9—C10—C11	119.3 (2)
C1—C2—H2	119.3	C9—C10—H10	120.4
C3—C2—H2	119.3	C11—C10—H10	120.4
C2—C3—C4	118.39 (19)	C10—C11—C12	119.6 (2)
C2—C3—C7	121.4 (2)	C10—C11—H11	120.2
C4—C3—C7	120.20 (19)	C12—C11—H11	120.2
O1—C4—C5	117.2 (2)	C13—C12—C11	121.7 (2)
O1—C4—C3	122.5 (2)	C13—C12—H12	119.2
C5—C4—C3	120.23 (19)	C11—C12—H12	119.2
C6—C5—C4	120.0 (2)	C12—C13—C14	118.2 (2)
C6—C5—H5	120.0	C12—C13—C15	120.3 (2)
C4—C5—H5	120.0	C14—C13—C15	121.5 (2)
C5—C6—C1	120.6 (2)	C9—C14—C13	119.5 (2)
C5—C6—H6	119.7	C9—C14—H14	120.2
C1—C6—H6	119.7	C13—C14—H14	120.2
O2—C7—C3	120.3 (2)	C13—C15—H15A	109.5
O2—C7—C8	119.8 (2)	C13—C15—H15B	109.5
C3—C7—C8	119.90 (19)	H15A—C15—H15B	109.5
C7—C8—H8A	109.5	C13—C15—H15C	109.5
C7—C8—H8B	109.5	H15A—C15—H15C	109.5
H8A—C8—H8B	109.5	H15B—C15—H15C	109.5
C7—C8—H8C	109.5	N2—N1—C9	114.0 (2)
H8A—C8—H8C	109.5	N1—N2—C1	113.3 (2)
H8B—C8—H8C	109.5	C4—O1—H1	102 (2)
			
C6—C1—C2—C3	1.1 (3)	C4—C3—C7—C8	176.5 (2)
N2—C1—C2—C3	−177.57 (18)	C14—C9—C10—C11	−2.0 (3)
C1—C2—C3—C4	−0.8 (3)	N1—C9—C10—C11	177.8 (2)
C1—C2—C3—C7	177.24 (19)	C9—C10—C11—C12	0.6 (3)
C2—C3—C4—O1	179.8 (2)	C10—C11—C12—C13	1.0 (4)
C7—C3—C4—O1	1.7 (3)	C11—C12—C13—C14	−1.3 (3)
C2—C3—C4—C5	−0.2 (3)	C11—C12—C13—C15	178.9 (2)
C7—C3—C4—C5	−178.3 (2)	C10—C9—C14—C13	1.8 (3)
O1—C4—C5—C6	−179.1 (2)	N1—C9—C14—C13	−178.01 (19)
C3—C4—C5—C6	0.9 (3)	C12—C13—C14—C9	−0.1 (3)
C4—C5—C6—C1	−0.5 (3)	C15—C13—C14—C9	179.7 (2)
C2—C1—C6—C5	−0.5 (3)	C10—C9—N1—N2	−177.6 (2)
N2—C1—C6—C5	178.1 (2)	C14—C9—N1—N2	2.2 (3)
C2—C3—C7—O2	−179.8 (2)	C9—N1—N2—C1	−179.99 (17)
C4—C3—C7—O2	−1.8 (3)	C2—C1—N2—N1	177.09 (19)
C2—C3—C7—C8	−1.4 (3)	C6—C1—N2—N1	−1.5 (3)

### Hydrogen-bond geometry (Å, °)

**Table:**

*D*—H···*A*	*D*—H	H···*A*	*D*···*A*	*D*—H···*A*
O1—H1···O2	0.84 (4)	1.78 (4)	2.567 (3)	156 (4)

### Table 2 Selected geometric parameters (Å, °) calculated with X-RAY, PM3 and DFT

**Table:**

Parameters	X-ray	PM3	DFT/B3LYP*
C4—O1	1.343 (3)	1.351	1.331
C7—O2	1.235 (3)	1.228	1.242
C7—C8	1.488 (3)	1.502	1.513
C13—C15	1.493 (4)	1.486	1.511
C1—N2	1.444 (3)	1.445	1.411
N1—N2	1.242 (3)	1.232	1.263
C9—N1	1.450 (3)	1.447	1.417
			
O2—C7—C8	119.8 (2)	120.465	118.986
O1—C4—C5	117.2 (2)	115.387	118.123
			
C7—C3—C4—O1	1.7 (3)	-0.016	0.002
C9—N1—N2—C1	-179.99 (17)	-179.965	-179.975
C2—C1—N2—N1	177.09 (19)	-178.543	179.996
C10—C9—N1—N2	-177.6 (2)	-172.651	179.997

*6-31G(d,p).

## References

[bb1] Bahatti, H. S. & Seshadri, S. (2004). *Coloration Technol.* **120**, 151–155.

[bb2] Becke, A. D. (1988). *Phys. Rev.* **A38**, 3098–100. 10.1103/physreva.38.3098 9900728

[bb3] Becke, A. D. (1993). *J. Chem. Phys.* **98**, 5648–5652.

[bb4] Catino, S. C. & Farris, R. E. (1985). *Concise Encyclopedia of Chemical Technology*, pp. 142–144. New York: John Wiley and Sons

[bb5] El-Ghamry, H., Issa, R., El-Baradie, K., Isagai, K., Masaoka, S. & Sakai, K. (2008). *Acta Cryst.* E**64**, o1673–o1674. 10.1107/S1600536808024239 PMC2960714 21201664

[bb6] Fadda, A. A., Etmen, H. A., Amer, F. A., Barghout, M. & Mohammed, K. S. (1994). *J. Chem. Technol. Biotechnol.* **61**, 343–349.

[bb7] Farrugia, L. J. (1997). *J. Appl. Cryst.* **30**, 565.

[bb8] Farrugia, L. J. (1999). *J. Appl. Cryst.* **32**, 837–838.

[bb9] Frisch, M. J., *et al.* (2004). *GAUSSIAN03* Gaussian Inc., Wallingford, CT, USA.

[bb10] Klaus, H. (2003). *Industrial dyes, chemistry, properties, applications*, pp. 20–35. New York: Wiley-VCH.

[bb11] Lee, C., Yang, W. & Parr, R. G. (1988). *Phys. Rev.* **B37**, 785–789. 10.1103/physrevb.37.785 9944570

[bb12] Schmidt, J. R. & Polik, W. F. (2007). *WebMO Pro.WebMO, LLC: Holland, MI, USA; available from* http://www.webmo.net.

[bb13] Sheldrick, G. M. (2008). *Acta Cryst.* A**64**, 112–122. 10.1107/S0108767307043930 18156677

[bb14] Stoe & Cie (2002). *X-AREA* and *X-RED* Stoe & Cie, Darmstadt, Germany.

[bb15] Taniike, K., Matsumoto, T., Sato, T., Ozaki, Y., Nakashima, K. & Iriyama, K. (1996). *J. Phys. Chem.* **100**, 15508–15516.

[bb16] Zollinger, H. (2003). *Color Chemistry*, 3rd revised ed. New York: Wiley-VCH.

